# In vitro activity of *Spirulina platensis* water extract against different *Candida* species isolated from vulvo-vaginal candidiasis cases

**DOI:** 10.1371/journal.pone.0188567

**Published:** 2017-11-30

**Authors:** Antonella Marangoni, Claudio Foschi, Matteo Micucci, Rogers Alberto Nahui Palomino, Tullia Gallina Toschi, Beatrice Vitali, Luca Camarda, Mara Mandrioli, Marta De Giorgio, Rita Aldini, Ivan Corazza, Alberto Chiarini, Roberto Cevenini, Roberta Budriesi

**Affiliations:** 1 Department of Specialized, Experimental, and Diagnostic Medicine (DIMES), Operative Unit of Clinical Microbiology, St. Orsola-Malpighi University Hospital, Alma Mater Studiorum-University of Bologna, Bologna, Italy; 2 Department of Pharmacy and Biotechnology (FaBit), Alma Mater Studiorum-University of Bologna, Bologna, Italy; 3 Department of Agricultural and Food Sciences (DiSTAL), Alma Mater Studiorum-University of Bologna, Cesena, Italy; Louisiana State University, UNITED STATES

## Abstract

The high incidence of vulvo-vaginal candidiasis, combined with the growing problems about azole resistance and toxicity of antifungal drugs, highlights the need for the development of new effective strategies for the treatment of this condition. In this context, natural compounds represent promising alternatives. The cyanobacterium *Spirulina platensis*, a blue-green alga, exhibits antimicrobial activities against several microorganisms. Nevertheless, only few data about the antifungal properties of *Spirulina platensis* are available and its potential toxic effects have not been largely investigated.

The aim of this study was to evaluate the in vitro activity of a fully-characterized water extract of *Spirulina platensis* against 22 strains of *Candida* spp. Prior to considering its potential topical use, we both investigated whether the extract exerted target activities on guinea pig uterine smooth muscle, and the impact of *Spirulina platensis* on the dominant microorganisms of the vaginal microbiota (i.e., lactobacilli), in order to exclude possible adverse events. By means of a broth microdilution assay, we found that the microalga extract possesses good antifungal properties (MIC: 0.125–0.5 mg/ml), against all the *Candida* species with a fungicidal activity. At the concentrations active against candida, *Spirulina platensis* did not modify the spontaneous basic waves pattern of uterine myometrium as underlined by the absence of aberrant contractions, and did not affect the main health-promoting bacteria of the vaginal ecosystem. Finally, we evaluated the selectivity index of our extract by testing its cytotoxicity on three different cell lines and it showed values ranging between 2 and 16.

Further in vivo studies are needed, in particular to evaluate the use of control-release formulations in order to maintain *Spirulina platensis* concentrations at anti-*Candida* active doses but below the toxic levels found in the present work.

## Introduction

Vulvovaginal candidiasis (VVC) represents the most frequent mucocutaneous mycosis caused by yeasts of the genus *Candida*. Although data about VVC incidence are incomplete, it is estimated that this condition represents a common problem worldwide, compromising the quality of life of many women [1–4].

Although the pathogenesis of symptomatic VVC remains undefined and debatable, it seems that, when the balance of the microbial local flora is altered, there is the creation of a suitable environment for *Candida* to proliferate and overgrow [5,6].

The therapeutic management of VVC is well-defined, nevertheless some issues remain open and not yet resolved, in particular for non-albicans *Candida* species being associated with reduced antifungal susceptibility, higher risk of recurrent candidiasis and very severe mucositis [1–2].

First, recurrent VVC represents an open challenge in term of therapeutic approach, since it could require prolonged maintenance-suppressive regimen of antifungal drugs [2]. In the second place, many studies have underlined the increasing prevalence of azole-resistant strains of *Candida albicans*, after prolonged maintenance of fluconazole treatment [7]. In the third place, non-albicans *Candida* strains isolated from vulvo-vaginal infections, as *Candida glabrata* and *Candida krusei*, show reduced sensitivity to fluconazole, requiring different drugs for their management [2]. Last, but not least, antifungal drugs have potential side-effects that limit their use in some patients [8].

The high incidence of vulvo-vaginal *Candida* infections together with the growing problems just mentioned above highlight the need for the development of new effective strategies for the prevention and therapy of these conditions.

In this context, the potential use of probiotic formulations based on different strains of *Lactobacillus spp*. has been investigated but, despite the promising results of some studies, further studies are indeed necessary to prove the effectiveness of probiotics for this condition [5,9].

Moreover, natural compounds are promising therapeutic alternatives because they tend to display fewer and lower intensity adverse reactions compared to commercial antifungal drugs [10].

Up to now, a large amount of secondary metabolites from natural extracts have been screened for their potential antimicrobial activity, but relatively few were found to be sufficiently active for humans [11,12].

Besides plants, in the recent years the search for cyanobacteria with antimicrobial activity has gained importance due to the growing concern about alarming increase in the rate of infection by multi-drug resistant microorganisms [13]. Various strains of cyanobacteria, are known to produce a wide variety of metabolites with different biological activities, such as antimicrobial and immunodulatory effects [13–16].

The cyanobacterium *Spirulina platensis*, a blue-green alga, has been used as a model organism in many studies on the cultivation of algal biomass as a source of proteins and chemicals [17]. Besides the high amount of proteins, *Spirulina* is characterized by the presence of different pigments such as chlorophylls, carotenes and phycobilins (phycocyanin, allo-phycocyanin and phycoerythrin) [18]. Many studies have examined the effects of the integration of *Spirulina* in the diet, particularly in relation to the chromophore phycocyanin. Indeed, it has been shown that this pigment displays a wide range of effects, such as neuroprotective, hepatoprotective, anti-inflammatory and anti-oxidative properties [17,19–22].

Moreover, different studies have shown that various extracts of *Spirulina platensis* can inhibit the replication of viruses through a calcium chelating sulfated polysaccharide [16,17] and exhibit antimicrobial activities against Gram-positive and Gram-negative bacteria [13,17]. Nevertheless little information about antifungal properties of *Spirulina platensis* is available and its potential toxic effects have not been largely investigated.

The aim of this study was to evaluate the in vitro activity of a full characterized water extract of *Spirulina platensis* against clinically isolated strains of *Candida spp* and ATCC reference strains.

Moreover, we investigated whether the extract exerts target activities on the uterine smooth muscle. In order to exclude a negative effect on the vaginal microbiota, we studied the impact of *Spirulina platensis* on lactobacilli, that dominate the vaginal niche of healthy women and represent endogenous defence factors [5,23–25]. Finally, the cytotoxicity of *Spirulina platensis* against epithelial and fibroblastic cells was evaluated.

## Materials and methods

### *Spirulina platensis* chemical characterization

*Spirulina platensis* water extract was kindly supplied by Alchemistry srl. (Cesena, Italy).

Phycobilins, carotenoids, total chlorophyll and fat content, as well as total fatty acid composition, were determined. Chemical composition data are the results of three independent determinations.

### Determination of phycobilins

*Spirulina platensis* (10 mg) was suspended in 5 mL phosphate-buffered saline (pH: 6.7) and extracted by performing cycles of freezing at -20°C and thawing at 4°C, followed by sonication in an ultrasonic bath for 5 minutes, as already described [26]. According to Lawrenz, three cycles of freezing and thawing alternated with periods of extraction of 48, 72 and 96 h in a refrigerator at 4°C were performed [27]. After centrifugation at 2879 × g for 20 min, the supernatant was spectrophotometrically evaluated at λ = 615, 652, 562 (Mod.V-550 Jasco Corporation, Tokyo, Japan, spectrophotometer). Chemical analysis description is reported in Supporting Information.

The quantification of phycobilins (phycocyanin, allo-phycocianin and phycoerythrin) was then calculated by applying the equations formulated by Siegelman and Kycia [28].

### Determination of chlorophyll and carotenoids

Five mg of *Spirulina platensis* were suspended in 20 mL of a solution of 80% acetone in water (v/v) with Ultra Turrax homogenizer, (T25 basic; IKA-WERKE, Staufen, Germany) at a speed of 17500 rpm for 1 min in an ice bath and then sonicated for 1 min. After 30 minutes of incubation in the dark, the sample was centrifuged at 2879 × *g* for 20 min and the clear supernatant was used for the spectrophotometric measures. The spectrophotometric determination of total chlorophyll and total carotenoids was performed at λ = 470, 645, 646, 652, 663 and was performed by applying the equations formulated by Arnon *et al*. and by Wellburn *et al*. [29,30]. Moreover, for a more accurate quantification of low concentrations of photopigments, the determination of chlorophyll was also performed as suggested by Jeffrey *et al*. (S1 File) [31].

### Extraction of the lipid fraction and chromatographic lipid profile

The lipid fraction was extracted following the method proposed by Folch [32], modified by Boselli [33], using as a solvent a mixture of chloroform methanol 2:1 (v/v) (S1 File).

### Determination of total fatty acids

The fatty acid composition was determined by gas chromatographic analysis, according to the method NGD C42-1976. A portion of lipid extract were methylated with diazomethane and subsequently the sample was dissolved in hexane and trans-methylated with 2 N KOH in methanol. The composition of total fatty acid methyl esters was determined by injecting 1 μL of the supernatant of the trans-methylated solution into a gas chromatograph. The analyses were performed using a GC 8000 Series gas chromatograph (Fisons Instruments) equipped with a fused silica capillary column RTX 2330 (Restek, Bellefonte, USA), stationary phase 90% bis-cyanopropyl-polysiloxane, 10% phenil-cyanopropyl-polysiloxane, 100 m length, 0.25 mm I.D., 0.2 μm film thickness. Split-splitless injection (1:60) was used and helium was the carrier gas. The temperature program was as follows: from 100°C holding 3°C min-1 up to 180°C maintained for 10 min., holding 3°C min-1 up to 240°C maintained for 30 min. The compounds were then detected with a flame ionization detector (FID). During the entire chromatographic run a constant pressure of 260 kPa was maintained, and the temperature of the detector and the injector were set at 240°C.

### Assessment of antifungal activity

*Candida* strains used in the present study were part of a broad collection including yeasts isolated from vaginal swabs submitted to the Microbiology Laboratory of St. Orsola University Hospital of Bologna for routine diagnostic procedures. In particular, 19 *Candida* isolates including species of 11 *Candida albicans*, 3 *Candida glabrata*, *1 Candida lusitaniae*, 1 *Candida tropicalis*, 1 *Candida krusei*, 1 *Candida parapsilosis* and 1 *Candida guillermondii*, were used. All the clinical isolates were coded to assure full anonymity. Moreover, three ATCC strains, commonly used for antifungal sensitivity testing, were included in the study (*C*. *albicans* ATCC-24433, *C*. *albicans* ATCC-90028 and *C*. *glabrata* ATCC-90030).

Candida strains were grown aerobically in Sabouraud dextrose (SD) medium (Oxoid, Basingstoke, Hampshire, UK) at 35°C and final identification at species level were performed with matrix-assisted laser desorption ionization-time of flight mass spectrometry (MALDI-TOF MS analysis (Bruker Daltonik GmbH, Leipzig, Germany).

The in vitro anti-*Candida* activity of *Spirulina platensis* extract was determined by broth microdilution assay for antifungal agents in accordance with the European Committee on Antimicrobial Susceptibility testing (EUCAST) guidelines (www.eucast.org) [34]. Starting from a stock solution of 1 gr/ml of *Spirulina platensis* extract, an initial dilution was prepared in distilled water. Each well of a 96-well flat bottom microdilution tray was inoculated with 100 μl of yeast suspension (1–5 × 10^5^ CFU/ml) and with 100 μl of *Spirulina platensis* extract, serially two-fold diluted in RPMI 1640 medium (Gibco, Thermo Fisher Scientific Inc., Waltham, Usa) buffered to pH 7.0 with 0.165 M 3-(N-morpholino)-propanesulfonic (MOPS) acid buffer and 2% glucose. In this way the final inocolum of yeast was 0.5–2.5 × 10^5^ CFU/ml and the final concentrations of microalga tested ranged from 16 mg/ml to 0.063 mg/ml.

The minimum inhibitory concentration (MIC) was considered as the lowest concentration of microalgae extract giving rise to an inhibition of growth of ≥ 50% of that of the extract-free control.

To determine the minimal fungicidal concentration (MFC) of *Spirulina platensis* extract, 50 μl of samples from wells exhibiting less than 50% of growth were subcultured onto SD agar plates and incubated at 35°C for 24/48 h. MFC was defined as the lowest drug concentration that showed either no growth or fewer than 3 colonies to obtain approximately 99 to 99.5% of killing activity [35].

*Candida* strains were tested with itraconazole and fluconazole and the MICs obtained were compared with the expected ones on the basis of data on MIC distribution, available on EUCAST website (www.eucast.org) [34].

A synergistic effect of Spirulina platensis extract with itraconazole and fluconazole was assessed, using a checkerboard test, as previously described [36]. All the experiments were conducted in triplicate.

### Functional contractility studies on guinea pig uterus

In order to exclude *Spirulina platensis* target activities on uterus spontaneous and induced contractility, functional studies on guinea pigs uterine smooth muscle were performed. Immediately after the sacrifice by cervical dislocation, the organs of the donor animals were excised and set up rapidly under a suitable resting tension in 15 ml organ bath containing Sund’s salt solution. Uterine horns strips were suspended in organ baths at an initial tension of 1 g in a Sund’s solution (mM): NaCl 154.0, KCl 5.63, CaCl_2_ 0.48, MgCl_2_ 0.98, NaHCO_3_ 5.95, glucose 2.78. Solutions were constantly warmed at 37°C and buffered to pH 7.4 by saturation with 95% O_2_ − 5% CO_2_ gas mixture.

#### Spontaneous contractility

The tracing graphs of spontaneous contractions were continuously recorded with the LabChart Software (AD Instruments, Bella Vista, New South Wales, Australia) using a force displacement transducer (FT 0.3, Grass Instruments Corporation). After an equilibration period (30–45 minutes) cumulative-concentration response curves of *Spirulina platensis* (0.1, 0.5, 1, 5 and 10 mg/mL) were constructed. For each concentration, 20 minutes time observation was done; the following parameters were evaluated considering a 5 minutes stationary period, immediately before the next concentration added for the cumulative curve (S2 File).

#### Induced contractility

Uterus strips were set up as above described. After the equilibration period, guinea pig uterus strips were contracted by washing in Sund’s containing 80 mM KCl (equimolar substitution of K^+^ for Na^+^). When the contraction reached a plateau, different concentrations of the SP extract (0.01–10 mg/mL) were added cumulatively allowing any relaxation to obtain an equilibrated level of force.

### Susceptibility of lactobacilli to *Spirulina platensis*

In order to assess the compatibility of *Spirulina platensis* towards the endogenous microbiota which plays a key role in human health, we investigated the activity of this compound against some representative species of vaginal symbiotic communities.

Several *Lactobacillus* strains isolated from human vaginal microbiota were used. Specifically, *L*. *crispatus* BC1, *L*. *crispatus* BC3, *L*. *gasseri* BC9, *L*. *gasseri* BC13, *L*. *vaginalis* BC15 and *L vaginalis* BC17 have recently been isolated from the vaginal ecosystem of healthy women [5]. Lactobacilli were grown in de Man, Rogosa and Sharpe (MRS) medium (Difco, Detroit, MI) supplemented with 0.05% L-cysteine. Bacterial cultures were incubated anaerobically for 24 h at 37°C in anaerobic jars supplemented with Anaerocult C (Merck, Milan, Italy). The inhibitory activity of *Spirulina platensis* was determined by the agar dilution method following the procedure defined by the National Committee for Clinical Laboratory Standards [37]. Briefly, a stock solution of 1 g/ml of *Spirulina platensis* in water was used to prepare MRS agar plates containing scalar concentrations of the microalgae (10, 5, 2.5, 1.25, 0.625, 0.3125 mg/ml). A bacterial suspension of 5 × 10^6^ CFU/ml was prepared from a broth culture in log phase growth. A volume of 20 μl was used to inoculate MRS plates in order to obtain a bacterial inoculum of 10^4^ CFU per plate. Plates were made in duplicate and incubated anaerobically at 37°C for 24 h. Results were read by comparing the number of colonies seeded on MRS plates without *Spirulina* extract (control plates) with the growth of lactobacilli grown on MRS plates supplementd with the microalga extract.

### Citotoxicity of *Spirulina platensis* extract

For the experiments the following three different cell lines were used: HeLa cells (ATCC CCL-2), an epithelial line derived from a cervix adenocarcinoma, HEL 299 (ATCC CCL-137), a fibroblast line derived from normal human lung, and, VK2/E6E7 (ATCC CRL-2616), an epithelial line derived from human vaginal mucosa. Cells were grown according to ATCC guidelines at 37°C.

Cells were seeded in 96-well microplates (30,000 cells/well), and cultured for 24 hours. Culture media were then replaced with media containing *Spirulina* extract, at concentrations ranging from 16 mg/ml to 0.063 mg/ml. After 1, 4, 8, and 24 h, the wells were stained with 0.5% crystal violet in 20% (v/v) methanol for 30 min. Following extensive washing with PBS, the incorporated dye was eluted by the addition of 50 μl of 0.1 M sodium citrate in 50% (v/v) ethanol (pH 4.2), and optical densities were read at 540 nm. The concentration resulting in 50% cell death compared with untreated controls (CC_50_) was calculated according to the Reed-Muench method [38, 39].

### Ethical statements

All animals (female guinea pigs 300–350 g of body weight; Charles Rivers Laboratories, Calco LC, Italy) were housed. and treated according to the directives on the protection of animals used for scientific purposes (Directive 2010/63/EU of the European Parliament and of the Council) and the WMA Statement on Animal Use in Biomedical Research. All procedures followed the guidelines of animal care and were approved by the Ethics Committee of the University of Bologna (Bologna, Italy) (Protocol 14/72/12).

### Statistical analysis

Statistical analyses of MICs/MFCs and functional contractility studies were performed by using an unpaired Student’s *t* test (GraphPad Prism 5.02 Software, San Diego California USA, www.graphpad.com).

The potency of *Spirulina platensis* extract defined as EC_50_ and IC_50_ was calculated from concentration-response curves (Probit analysis using Litchfield and Wilcoxon [40] or GraphPad Prism 5.02 Software).

A *P* value <0.05 was considered significant.

## Results

### Chemical characterization

Phycobilins, carotenoids, total chlorophyll and fat content of *Spirulina platensis* extract are shown in details in Table 1.

**Table 1:** Chemical composition of *Spirulina platensis* water extract.

*Spirulina platensis* water extract components		Concentration
Phycobilins	Phycocyanin	8.38 ± 0.89 mg/100 mg
	Allo-phycocyanin	2.48 ± 0.60 mg/100 mg
	Phycoerythrin	1.99 ± 0.62 mg/100 mg
Carotenoids		1.97 ± 0.12 mg/g
Total chlorophyll		8.61 ± 0.48 mg/g^a^ 8.48 ± 0.48 mg/g^b^ 8.18 ± 0.49 mg/g^c^ 11.21 ± 0.51 mg/g^d^
Fat		7.92 ± 0.16 w%^e^

^a^Value determined by applying the equation number 1, proposed by Arnon,1949 [29]

^b^Value determined by applying the equation number 2, proposed by Arnon,1949 [29]

^c^Value determined by applying the equation proposed by Wellburn, 1994 [30]

^d^Value determined by applying the equation proposed by Jeffrey e Humphrey, 1975 [31]

^e^w % indicates weight fraction percentage

**Fig 1:**
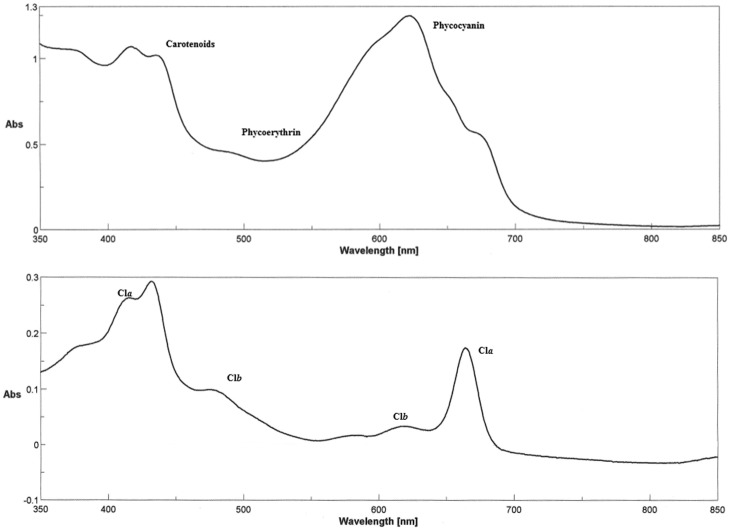
Chromatographic profile of the *Spirulina platensis*. Upper panel: spectrum (350 to 850 nm) of the *Spirulina platensis* extract analyzed. Lower panel: spectrum (350 to 850 nm) of the 80% acetone extract of *Spirulina platensis*, used for the quantification of the total chlorophylls. Chlorophyll A (Cla) and B (Clb) profile in the *Spirulina platensis* analyzed (see Method section).

Concentrations are expressed in mg/100 mg for phycobilins and carotenoids, in mg/g for chlorophylls and in w % for fat content. Briefly, the phycocyanin appeared to be the most represented pigment in the group of phycobilins (phycocyanin, allo-phycocianin, phycoerythrin) whereas the different algorithms applied for the calculations of chlorophyll content showed comparable results (Table 1). The spectrophotometric analysis of *Spirulina platensis* extract and the spectrum used for the quantification of total chlorophyll are shown in Fig 1 upper and lower panel, respectively.

A modest amount of fat (7.92%) was found in the microalga extract and when the lipid fraction was separated through TLC, the presence of various classes of compound was noticed including hydrocarbons, free fatty acids, sterols, monoglycerides, diglycerides and triglycerides. In S1 Fig, showing total lipid gas chromatographic profile, the contribution of triglycerides in the overall lipid content was low, whereas hydrocarbons represented the most relevant fraction.

In the extract analyzed, the composition of fatty acids methyl esters highlighted a significant value of polyunsaturated fatty acids (38.6%), among which the 15.8% was represented by the C18:3 γ-linoleic acid. On the other side, palmitic acid was the most represented saturated fatty acid (49.2%). The composition of fatty acids methyl esters obtained by gas chromatography is shown in S1 Table.

### Antifungal activity

The results of anti-*Candida* activity of *Spirulina platensis* are shown in details in Table 2. MIC and MFC values showed no differences between the three replicates of each test.

**Table 2:** MIC and MFC values of microalga extract for *Candida* strain included in the study.

Strain	*Candida* species	S. platensis MIC^a^ (mg/mL)	S. platensis MFC^b^ (mg/mL)	Itraconazole MIC^a^ (mg/L)	Fluconazole MIC^a^ (mg/L)
**1**	*C*. *albicans*	0.25	0.25	0.015	0.25
**2**	*C*. *albicans*	0.25	0.25	0.015	0.125
**3**	*C*. *albicans*	0.25	0.25	0.015	0.125
**4**	*C*. *albicans*	0.25	0.25	0.015	0.06
**5**	*C*. *albicans*	0.125	0.125	0.015	0.125
**6**	*C*. *albicans*	0.25	0.25	0.03	0.25
**7**	*C*. *albicans*	0.25	0.25	0.015	0.125
**8**	*C*. *albicans*	0.25	0.25	0.015	0.125
**9**	*C*. *albicans*	0.25	0.25	0.03	0.25
**10**	*C*. *albicans*	0.25	0.25	0.015	0.06
**11**	*C*. *albicans*	0.25	0.25	0.03	0.125
**12**	*C*. *glabrata*	0.5	0.5	0.5	8
**13**	*C*. *glabrata*	0.5	0.5	0.5	8
**14**	*C*. *glabrata*	0.5	0.5	1	16
**15**	*C*. *lusitaniae*	0.125	0.125	0.015	0.5
**16**	*C*. *tropicalis*	0.125	0.125	0.03	0.25
**17**	*C*. *krusei*	0.125	0.125	0.25	32
**18**	*C*. *parapsilosis*	0.5	0.5	0.03	0.5
**19**	*C*. *guillermondii*	0.5	0.5	0.25	4
**ATCC-24433**	*C*. *albicans*	0.25	0.25	0.015	0.125
**ATCC-90028**	*C*. *albicans*	0.25	0.25	0.03	0.25
**ATCC-90030**	*C*. *glabrata*	0.5	0.5	1	16

^a^MIC = Minimum Inhibitory Concentration.

^b^MFC = Minimal Fungicidal Concentration.

The MIC and MFC values of microalga extract for *Candida* strain ranged from 0.125 to 0.5 mg/ml. Notably, for all the *Candida* strains analyzed during the study, the MFC corresponded exactly to MIC values.

**Fig 2:**
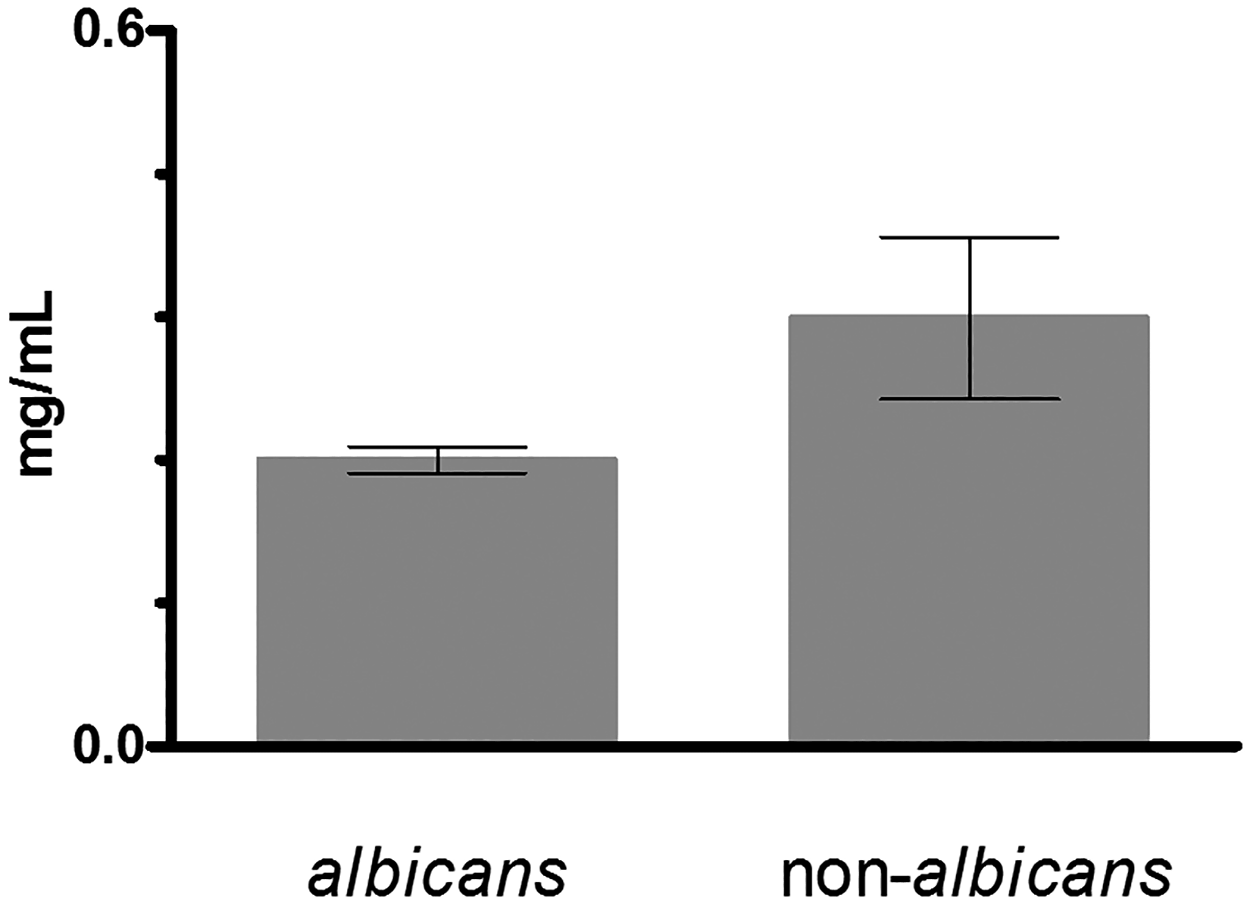
MIC values of *Spirulina platensis* against *C*. *albicans* and *C*. *non-albicans* strains. The columns represent the MIC values of *C*. *albicans* (13 strains) and *C*. *non-albicans* (9 strains), respectively. Data are reported as mean values ± Standard Deviations (SD) calculated among the different isolates of each group.

The strains belonging to *Candida glabrata*, *Candida parapsilosis* and *Candida guillermondii* species showed the higher MIC and MFC values. Strains of the remaining species were characterized by variable MIC and MFC values. In Fig 2 a comparison between the MICs of *C*. *albicans* strains vs. *C*. *non-albicans* strains is reported. The *C*. *albicans* isolates were characterized by significant lower MICs than *C*. *non-albicans* strain (*P* = 0.0189).

When the MICs of itraconazole and fluconazole for *Candida* strains were verified by the microdilution assay, results were comparable to the EUCAST data about MIC distribution.

No synergistic effect was noticed between *Spirulina platensis* extract and traditional antifungal drugs (itraconazole and fluconazole), being the fractional inhibitory concentration index (FICI) = 2 in both cases, thus demonstrating an indifferent effect.

### Uterus contractility

**Fig 3:**
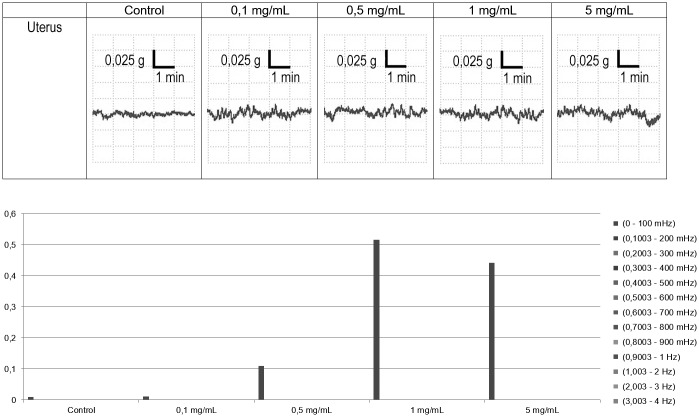
Uterus contractility. Upper panel: experimental original recording showing a typical concentration-response effects of *Spirulina platensis* extract on spontaneous uterus contractility. Lower panel: spontaneous contractility absolute powers observed in the same experiment.

The effect of *Spirulina platensis* on the uterine horns spontaneous contractility was tested. The spontaneous uterus contractility showed a typical panel of waves. The control represents the basal activity of the uterus, without any active substance; *Spirulina platensis* did not modify the spontaneous rhythmic basic phasic pattern contractions (tone) of uterine horns preparations up to the highest concentration tested as underlined by the absence of aberrant contractions as shown in Fig 3. All over the trace during the cumulative concentration-response curve a significant increase of the low frequency (0,1003-200 mHz) waves occurred along the *Spirulina platensis* concentration.

**Fig 4:**
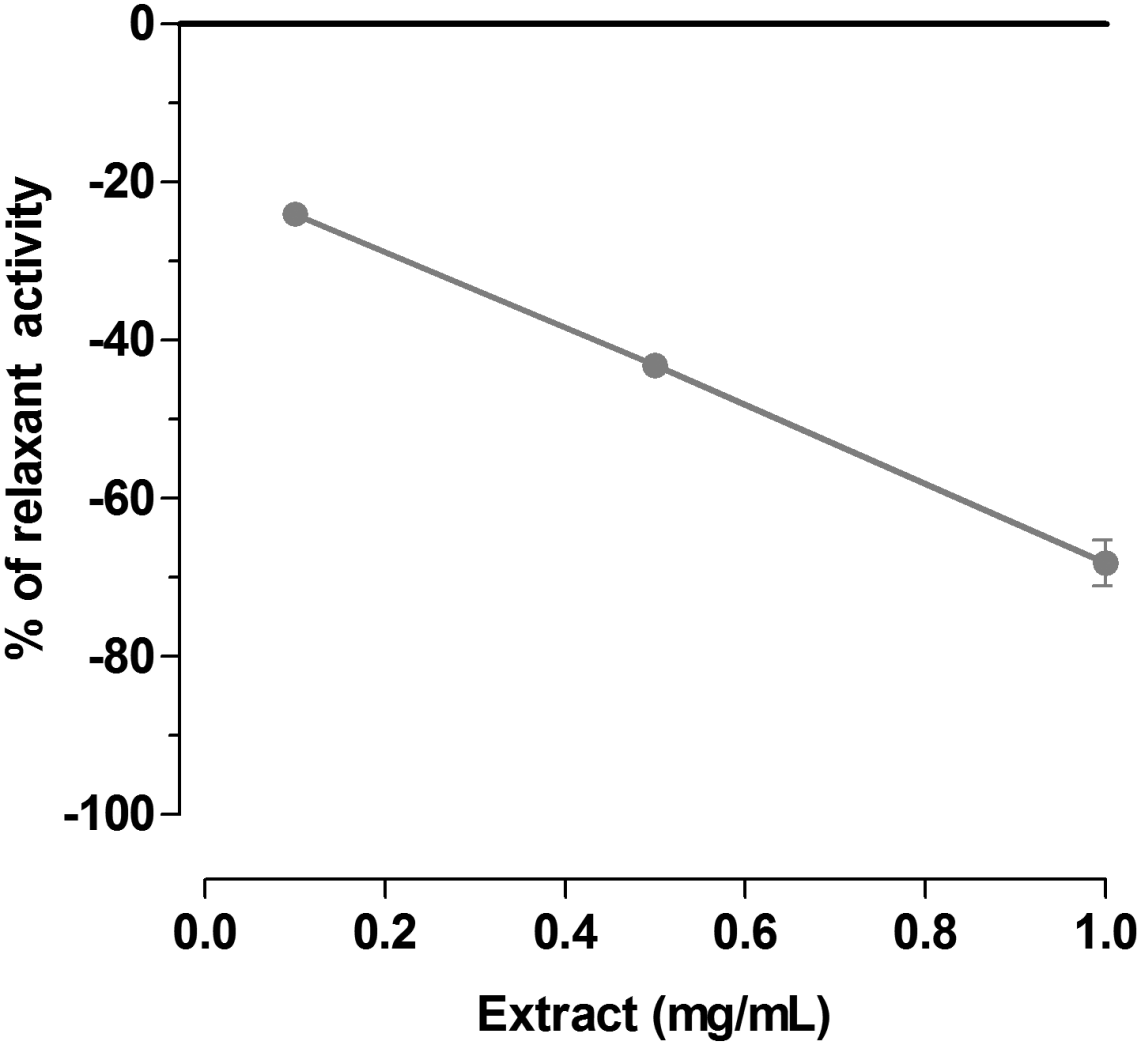
Cumulative concentration-response curves for *Spirulina platensis* extract on K^+^ (80 mM) depolarized guinea-pig uterus. Each point represents the percent inhibition to maximal contraction induced by 80 mM K^+^ assumed as 100% (0 in figure). Each point is the mean ± SEM of four-six experiments. Where error bars are not shown, these are covered by the point.

*Spirulina platensis* was also tested for its spasmolytic activity against L-Type calcium channels by experiments performed on isolated uterine horns 80 mM K^+^-depolarized. *Spirulina platensis* induced concentration-dependent spasmolytic activity (Fig 4). The intrinsic activity is about 60% at 1 mg/mL and the potency is 0.19 mg/ml (c.l. 0.075–0.25).

### Compatibility of *Spirulina platensis* with health-promoting bacteria of vaginal microbiota

All *Lactobacillus* strains grew in the presence of *Spirulina platensis* up to the highest concentration tested (10 mg/ml), with no statistical differences between the number of colonies grown on control MRS plates and MRS plates supplemented of microalga extract, thus suggesting that this extract does not affect the main health-promoting components of vaginal ecosystem.

### Cytotoxicity of *Spirulina platensis* extract

We evaluated the cytotoxicity of the *Spirulina* extract upon three different lines: HeLa, HEL 299 and VK2/E6E7 cells with the MTT assay. The CC_50_ was the same for all the lines: 2 mg/mL at 1, 4 and 8 h, and 1 mg/mL at 24 h. Since the IC_50_ (the minimum concentration for inhibiting 50% of the pathogen) found in this study ranged between 0.125 mg/mL and 0.5 mg/mL, the selectivity index (SI) calculated as the ratio of CC_50_ to IC_50_, was therefore comprised between 2 and 16.

## Discussion

Natural compounds are promising therapeutic alternatives compared to traditional antifungal drugs, because they present smaller and lower adverse reactions and they tend to show better cost-benefit ratios [10].

The cyanobacterium *Spirulina platensis* possesses various biological and nutritional activities with bio-modulatory, immuno-modulatory functions and antimicrobial properties [41–44]. Taken together these results provide important information for the potential application of *Spirulina platensis* in the treatment of systemic candidiasis [45].

*Spirulina platensis* has been found to be active against several enveloped viruses through the inhibition of their replication [16,39] and *Spirulina platensis* methanol extracts show potent antimicrobial activity especially against *Streptococcus faecalis*, *Staphylococcus epidermidis* and *Candida albicans* [17].

Similarly, *Spirulina platensis* methanolic extract exhibits broad spectrum anti-bacterial activity for *Pseudomonas aeruginosa* and *Salmonella typhi* [13].

The chemical characterization of *Spirulina platensis* water extract showed comparable results with other researchers, regarding the amounts of phycocyanin [46–49], carotenoids [49, 50] and total chlorophyll levels [51, 52]. The slight differences found from one to another study can probably be due to the extraction protocol used and to the chemical nature of the extract. The total amount of fat and the composition of lipid fraction agrees with previous reported data [53, 54].

We evaluated the anti-*Candida* activity of *Spirulina platensis* extract by a microdilution assay, testing 22 strains of *Candida* belonging to different species and isolated from vaginal swabs or obtained from ATCC. At our knowledge, this is the first study that investigate the antifungal activity of this microalga against a wide panel of yeasts of clinical origin and that evaluate its real MIC and MFC values. Indeed, Ozdemir *et al*. studied only an ATCC strain of *Candida albicans* by a simple disk diffusion method, measuring the diameters of zones of growth inhibition [17].

Our results showed that *Spirulina platensis* extract is active against all *Candida* strains tested with MIC values ranging from 0.125 to 0.5 mg/ml. Since MFC and MIC values were the same, we demonstrated therefore that *Spirulina platensis* exerts its anti-*Candida* activity in a fungicidal way.

In this context, the finding that phycocyanin appears to be the most represented phycobilin [23] in our extract, is of particular interest. Effectively, it has been shown that phycocyanin displays significant anti-inflammatory effects through the inhibition of COX-2 and lipo-oxygenase enzymes, with reduction in prostaglandins and leukotrienes levels [19]. In case of severe mucositis, such as VVC due to non-albicans *Candida* species, the anti-fungal activity together with the anti-inflammatory properties of *Spirulina platensis* extract could probably exert a combined useful effect.

Thinking about a potential use of the microalga extract as a topical agent for VVC treatment and in order to exclude toxic effects, at first, we performed functional studies on the uterine smooth muscle contractility. The degree of contractility/relaxation of the uterus depends on the excitability level of the smooth muscle cells of the myometrium, with the interaction of hormonal, biochemical and neurovegetative factors. Indeed, we evaluated the basal myometrium contractility in guinea pig uterus smooth muscle during ex vivo experiments, without any connection to the hormonal state, considering the use of *Spirulina platensis* independent from the menstrual phase.

Myometrium undergoes rhythmic oscillations, which have been termed ‘slow waves’ [55]: in basal conditions, they were not altered by the presence of *Spirulina platensis*, even though the myometrium contractile tone increased together with the microalga concentration. Our results suggest that *Spirulina platensis*, at the concentrations active against *Candida* strains, does not alter the spontaneous contractility of uterine smooth muscle.

Calcium channel modulators are multidrug resistance reverters [56]. The mild calcium modulating effect may contribute increasing the antifungal effect of *Spirulina platensis*, as it was shown that Verapamil enhances the effect of antifungal drugs against *Candida albicans* biofilm [57]. Therefore, we have tested the extract on a specific model ex vivo in order to evaluate its effects on calcium channels. In guinea pig uterus K^+^ 80 mM depolarized, *Spirulina platensis* induced a spasmolytic potency at a concentration similar to the one corresponding to its MIC.

Globally, the microalga extract combines a significant antifungal activity to a mild calcium modulator effect, which can be useful in case of infections due to resistant microorganisms.

Moreover, we assess the compatibility of *Spirulina platensis* towards the vaginal endogenous microbiota, evaluating the effects of the microalga towards several *Lactobacillus* strains of vaginal origin.

Lactobacilli are representative microbial species of vaginal symbiotic communities and play a fundamental role in promoting and maintaining human health [23]. A disruption on the balance of normal microbiota can result in different pathological conditions, as bacterial vaginosis and exogenous or endogenous infections [58, 59]. Our results suggest that *Spirulina platensis*, at the concentrations active against *Candida* strains, does not affect the main health-promoting components of the human ecosystems. These results seem to be in contrast with the anti-bacterial effects of *Spirulina platensis*, described by several authors [13, 17]. One explanation could lie in the different types of *Spirulina* extract used (alcoholic vs aqueous). Effectively, as already reported by Ozdemir and by Kaushik, various chemical extracts (methanol, dichloromethane, petroleum ether, ethyl acetate extracts) could display significant different levels of anti-bacterial activity [13, 17]. Moreover, the different bacterial species tested in this study (lactobacilli) compared with the other reports (staphylococci, enterobacteria) could explain the completely different effect of the same concentrations of *Spirulina platensis* extract. We can speculate that the selective anti-*Candida* activity showed by the microalga extract could be the result of a specific mechanism of action, related to the inhibition of fungal components, absent in bacterial cells (i.e. fungal wall).

Finally, we evaluated the in vitro cytotoxicity effects of *Spirulina platensis* extract on three different cell lines (HeLa, HEL 299 and VK2/E6E7cells). The selectivity index ranged between 2 and 16, being lower than another extract used by Chen [36]. He and his colleagues found an extremely safe and well-tolerated cold water extract of *Spirulina platensis*, with high selectivity index in cellular toxicity studies, when tested against influenza virus replication and plaque formation. Indeed, C-phycocyanin reduced apoptosis in HeLa cells [60]. These apparent partially discordant findings could potentially be ascribed to the high concentration necessary to the antifungal activity. In this context, several aspects should be taken into account. In particular, we are fully aware that our citotoxicity results merely derive from in vitro studies. Considering that the vaginal mucosa possesses peculiar anatomical features (i.e. stratified squamous epithelium, mucus production), in vivo results could be completely different. For that reasons, animal studies are necessary to confirm or exclude the citotoxicity effects found in cell lines in vitro, and further information is needed for the use of *Spirulina platensis* extract as a topical agent in the clinical practice for VVC treatment.

Moreover, the possibility of the use of *Spirulina platensis* extract control-release formulations most importantly can maintain the microalga concentrations at anti-*Candida* effective doses, but below the cytotoxic levels, avoiding potential toxic effects [60].

## Conclusions

In conclusion, to our knowledge this is the first report about the in vitro-activity of a water extract of *Spirulina platensis* against several vaginal isolates of different *Candida* species. The good anti-fungal properties, together with the absence of negative effects on the endogenous microbiota and on the spontaneous motility of uterine smooth muscle, could potentially allow the use of this extract as an alternative approach to topical antifungal agents for VVC treatment.

Further studies are needed to elucidate the mechanisms involved in the selective anti-fungal effect, to characterize the single fractions to find the chemiotypes responsible of the antifungal activity, and to assess the activity and the potential toxicity of *Spirulina platensis* extract in vivo, in particular using control-release formulations [61].

## Supporting information

S1 File Materials and methods. *Spirulina platensis* chemical characterization.
(DOCX)

S2 File In vitro contractility functional assays. (DOCX)

S1 Fig Total lipid gas chromatographic profile of Spirulina platensis, obtained after extraction with Folch method. (DOCX)

S1 Table Composition of fatty acids methyl esters by gas chromatography. The total fatty acids is expressed as the percentage of the corresponding methyl esters obtained from fatty substance extracted.
(DOCX)
