# Supplementary material for: In vitro activity of Spirulina platensis water extract against different Candida species isolated from vulvo-vaginal candidiasis cases
Source: PLoS One. 2017 Nov 30;12(11):e0188567. doi: 10.1371/journal.pone.0188567 (PMC5708745; doi:10.1371/journal.pone.0188567)
Supplement: S1 File — Spirulina platensis chemical characterization. (DOCX) [file pone.0188567.s001.docx]

**S1 Materials and methods**

***Spirulina platensis* chemical characterization**

***Determination of phycocianine***

The sample (10 mg) was suspended in 5 mL of phosphate buffer pH = 6.7 and extracted by performing cycles of freezing at -20 °C and thawing at + 4 °C, as previously described by Bennett and Bogorad [1], but each cycle was followed by sonication in an ultrasonic bath for 5 minutes, the method comprising cycles of freezing and thawing was already described by Bennett and Bogorad [1]. In particular three cycles of freezing and thawing were interspersed with periods of extraction of 48, 72 and 96 h in a refrigerator at +4 °C, as indicated by Lawrenz [2] given that, unlike the cryptophytes, cyanobacteria require a time up to 96 h for the complete extraction of phytopigments. The quantification of phycobilins was then calculated by applying the equation formulated by Siegelman and Kycia [3]:

Phycocyanin -PC (mg/mL) =[ Absʎ615 – (0.474 * Absʎ652)]/5.34

Allo-phycocyanin -APC (mg/mL) =[ Absʎ652 – (0.208 * Absʎ615)]/5.09

Phycoerythrin -PE (mg/mL) =[ Absʎ562 –2.41(PC) – 0.849*(APC)]/9.62

where:

PC, APC, PE are the respective concentrations of Phycocyanin, Allophycocianin, Phycoerythrin expressed in mg/mL. while Absʎ615, Absʎ652, Absʎ562 the absorbance values measured at wavelengths of 615, 652 and 562 nm in phosphate buffer.

***Determination of chlorophyll and carotenoids.***

For a more accurate quantification of low concentrations of photopigments, the determination of chlorophyll was also performed as suggested by Jeffrey *et al*. [4]. Briefly, 5 mg by spectrophotometric determination at aʎ=630,647,664, against a blank of aceton/water 90% (Mod.V-55, Jasco Corporation, Tokyo, Japan spectrophotometer) was suspended in a solution of 90 % acetone in water, as already reported by Parsons [5] and treated with Ultra Turrax homogenizer at a speed of 17500 rpm for 1 minute in an ice bath, then subsequently sonicated for 1 min. After 30 minutes of incubation in the dark, the sample was centrifuged at 2879 × *g* for 20 minutes and the clear supernatant was used for the spectrophotometric reading. The determination of chlorophyll was done by applying the equation of Jeffrey *et al*. [4].

*Extraction of the lipid fraction and chromatographic lipid profile*. After 1g homogenization with Ultra Turrax, the lipid extract was filtered and the filtrate was mixed thoroughly with a 1 M KCl solution and left overnight at 4°C, to obtain phase separation. The lower phase was collected and dried with a rotary evaporator. The fat content was determined gravimetrically. For the study of the lipid fraction a separation on a silica TLC plate was performed. A portion of the lipid extract has been deposited on TLC plate of silica gel and then eluted with a mixture of *n*-hexane-ether (65:35 v/v) in the developing chamber at a temperature of 4° C. The plate was finally developed by spraying the surface with a solution of phosphomolybdic acid; the detection of the analytes was then obtained placing the plate in an oven at 120° C, for 10 minutes.

In order to characterize its composition, the extracted lipid fraction was subjected to gas chromatographic analysis, by injecting a suitable amount of lipid extract into the gas chromatograph [6-10]. The analyses were performed by using a GC 8000 Series gas chromatograph (Fisons Instruments, Rodano (MI), Italy) equipped with a fused silica capillary column SE52, stationary phase 5% diphenyl-95% dimethyl polysiloxane, 10 m length, 0.25 mm I.D., 0.1 µm film thickness. Split-splitless injection (1:50) was used and helium (0.8 mL min-1) was the carrier gas. The temperature was programmed from 100 to 350 °C at 5°C min-1.

The compounds were then detected by a flame ionization detector (FID). During the entire gas chromatographic run the flow of helium was maintained constant, and the temperature of the detector and the injector were set at 350° C.

**References**

1. Bennett A and Bogorad L The Journal of Cell Biology Vol. 58, 1973 pages 419 -435.

2. Lawrenz E, Fedewa EJ, Richardson TL.. Extraction protocols for the quantification of phycobilins

in aqueous phytoplankton extracts. J Appl Phycol. 2011; 23:865 – 871.

3. Siegelman HW, Kycia JH. Algal biliproteins. In: Hellebust JA, Craigie JS (eds) Handbook of phycological methods, physiological and biochemical methods, Cambridge University Press, Cambridge, 1978, pp. 71–79.

4. Jeffrey SW, Humphrey GF. New spectrophotometric equations for determining chlorophyll a, b, c 1 and c 2 in higher plants and natural phytoplankton. Bioch Physiol Pﬂanz. BPP1975;165: 191–194.

5. Parsons TR, Strickland JDH. Particulate organic matter III. I. Pigment analysis III, Determination of Phytoplankton pigments. J. Fish.Res. 1965;18: 117-127.

6. Colla LM, Bertolin TE, Vieira Costa JA. Fatty Acids Profile of Spirulina platensis Grown Under Different Temperatures and Nitrogen Concentrations. Z Naturforsch. 2004; 59c:55-59.

7. Ambrozova JV, Misurcova L, Vicha R, et al.. Influence of extractive solvents on lipid and fatty acids content of edible freshwater algal and seaweed products, the green Microalga Chlorella kessleri and the Cyanobacterium Spirulina platensis. Molecules. 2014; 19:2344–60.

8. Babadzhanov AS, Abdusamatova N, Yusupova FM, et al. Chemistry of Natural Compounds. 2004; 40, No. 3,.

9. Sajilata MG, Singhal MS, Kamat MY. Fractionation of lipids and purification of γ-linolenic acid (GLA) from Spirulina platensis. Food Chemistry. 2008; 109:580–586.

10. Nichols BW, Wood BJB. The Occurrence and Biosynthesis of gamma-Linolenic Acid in a Blue-Green Alga, Spirulina platensis. Lipids. 1967; 3:46-50.
