# Supplementary material for: In vitro activity of Spirulina platensis water extract against different Candida species isolated from vulvo-vaginal candidiasis cases
Source: PLoS One. 2017 Nov 30;12(11):e0188567. doi: 10.1371/journal.pone.0188567 (PMC5708745; doi:10.1371/journal.pone.0188567)
Supplement: S2 File — (DOCX) [file pone.0188567.s002.docx]

**S2 Materials and methods**

**In vitro spontaneous contractility functional assays.**

At the end of each single concentration (20 minutes exposure), the following parameters were evaluated considering a 5 minutes stationary period:

- the Basal Spontaneous Motor Activity (BSMA), calculated as the mean force value (g) expressed as % variation from the control;

- the Basal Contraction Amplitude (BCA), calculated as the mean value of the differences between each force peak and the basal level of spontaneous phasic contraction (% variation from the control) considering the most visible peaks on the recordings.

- the spontaneous contractions rates through a standard Fast Fourier Transform (FFT) analysis and the evaluation of the percentage powers of the following frequency bands of interest (expressed in Hz): [0.0,0.1] ]0.1,0.2] ]0.2,0.3] ]0.3,0.4] ]0.4,0.5] ]0.5,0.6] ]0.6,0.7] ]0.7,0.8] ]0.8,0.9] ]0.9,1.0] ]1.0,2.0] ]2.0,3.0] ]3.0,4.0] ]4.0,5.0] ]5.0,6.0] ]6.0,7.0] ]7.0,8.0] ]8.0,9.0] ]9.0,10.0].

- All the calculations were performed in a post-processing phase with the Lab Chart Software. In order to avoid errors due to the presence of artifacts, the period of analysis was chosen by a skilled operator.

Functional contractility studies statistical analysis: Higher than 10% percent variations of each range were considered statistically significant.
