# Supplementary material for: In vitro activity of Spirulina platensis water extract against different Candida species isolated from vulvo-vaginal candidiasis cases
Source: PLoS One. 2017 Nov 30;12(11):e0188567. doi: 10.1371/journal.pone.0188567 (PMC5708745; doi:10.1371/journal.pone.0188567)
Supplement: S1 Fig — (DOCX) [file pone.0188567.s003.docx]

**
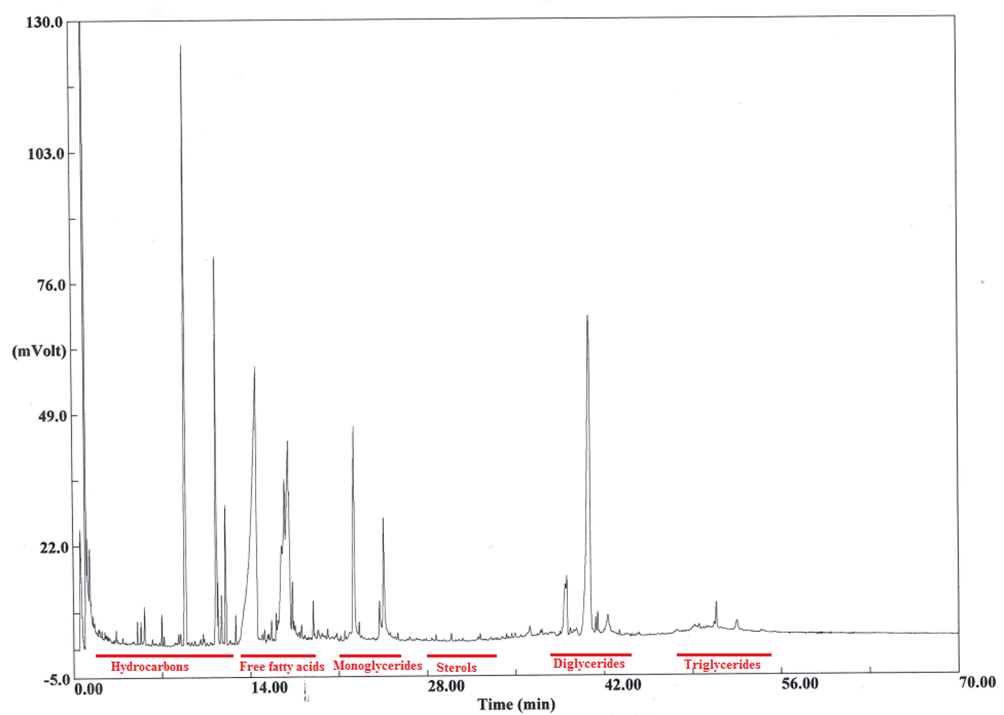
**

**S3 Fig. Total lipid gas chromatographic proﬁle of *Spirulina Platensis*, obtained after extraction with Folch method.**
