# Supplementary material for: In vitro activity of Spirulina platensis water extract against different Candida species isolated from vulvo-vaginal candidiasis cases
Source: PLoS One. 2017 Nov 30;12(11):e0188567. doi: 10.1371/journal.pone.0188567 (PMC5708745; doi:10.1371/journal.pone.0188567)
Supplement: S1 Table — The total fatty acids is expressed as the percentage of the corresponding methyl esters obtained from fatty substance extracted. (DOCX) [file pone.0188567.s004.docx]

**S1 Table. Composition of fatty acids methyl esters (FAME) by gas chromatography**

| **FAME** | **FAME**  **%^a^** | **Colla**  **[6]** | **Ambrozova**  **[7]** | **Babadzhanov**  **[8]** | **Sajilata**  **[9]** | **Nichols**  **[10]** |
| --- | --- | --- | --- | --- | --- | --- |
| **C8:0** | 0.15 |  |  |  |  |  |
| **C10:0** | 0.06 |  | nd-0.11 |  |  |  |
| **C12:0** | 0.01 |  | nd-0.21 | 0.4 |  |  |
| **C12:1** | 0.02 |  |  |  |  |  |
| **C14:0 Iso** | 0.02 |  |  |  |  |  |
| **C13:1** | 0.04 |  |  |  |  |  |
| **C14:0** | 0.21 |  | 0.46-0.56 | 0.8 | 4.38 |  |
| **C15:0 Iso** | 0.16 |  |  |  |  |  |
| **C15:0 AI** | 0.02 |  |  |  |  |  |
| **C14:1** | 0.01 |  |  |  |  |  |
| **C15:0** | 0.04 |  | nd-0.1 |  |  |  |
| **C16:0** | 49.29 | 44.29-48.27 | 53.02-61.06 | 44.9 | 53.09 | 43.4 |
| **C16:1 t** | 0.06 |  |  |  |  |  |
| **C17:0 Iso** | 0.06 |  |  |  |  |  |
| **C16:1 c7 (n9)** | 1.27 |  |  |  |  |  |
| **C16:1 n7** | 3.26 | 1.67-2.83 | 2.59-3.36 | 2.3 |  | 9.7 |
| **C17:0 AI** | 0.02 |  |  |  |  |  |
| **C17:0** | 0.15 |  | 0.27-0.28 | 1.2 |  |  |
| **C17:1** | 0.42 |  | nd-0.50 |  |  |  |
| **C18:0** | 0.87 | 0.89-1.57 | 1.40-4.75 | 2.2 | 1.8 | 2.9 |
| **Σ C18:1 trans** | 0.30 |  | nd-0.08 |  |  |  |
| **Σ C18:1 cis** | 3.68 | 6.84-9.29 | 2.79-5.29 | 10.1 | 18.37 | 5 |
| **C18:2cis-trans** | 0.37 |  |  |  |  |  |
| **C18:2** | 22.08 | 11.93-14.68 | 16.17-18.42 | 11.1 | 6.57 | 12.4 |
| **C20:0** | 0.05 |  | nd-0.06 |  |  |  |
| **C18:3 γ linol.** | 15.86 | 18.34-20.92 | 10.44-17.36 | 17.1 | 15.8 | 21.4 |
| **C18:3** | 0.03 |  | nd |  |  |  |
| **C20:1** | 0.05 |  | nd |  |  |  |
| **C20:2** | 0.13 |  | nd-0.11 |  |  |  |
| **C22:0** | 0.01 |  | nd |  |  |  |
| **C20:3 γ linol.** | 0.16 |  | nd-0.15 |  |  |  |
| **C20:4+C22:1** | 0.05 |  | nd |  |  |  |
| **Altri** | 1.09 |  |  | 9.9 |  |  |
| **Σ SFA** | 51.13 |  | 59.47-63.18 |  |  |  |

^a^The total fatty acids is expressed as the percentage of the corresponding methyl esters obtained from fatty substance extracted.
